# Specificity of emotion sequences in borderline personality disorder compared to posttraumatic stress disorder, bulimia nervosa, and healthy controls: an e-diary study

**DOI:** 10.1186/s40479-017-0077-1

**Published:** 2017-12-21

**Authors:** Tobias D. Kockler, Wolfgang Tschacher, Philip S. Santangelo, Matthias F. Limberger, Ulrich W. Ebner-Priemer

**Affiliations:** 1 Karlsruhe Institute of Technology, Mental mHealth Lab, Karlsruhe, Germany; 2 University of Bern, University Hospital of Psychiatry, Bern, Switzerland

**Keywords:** Borderline personality disorder, Ecological momentary assessment, Emotion, Affective dysregulation

## Abstract

**Background:**

Patients with borderline personality disorder (BPD) exhibit dysregulated emotion sequences in daily life compared to healthy controls (HC). Empirical evidence regarding the specificity of these findings is currently lacking.

**Methods:**

To replicate dysregulated emotion sequences in patients with BPD and to investigate the specificity of the sequences, we used e-diaries of 43 female patients with BPD, 28 patients with posttraumatic stress disorder (PTSD), 20 patients with bulimia nervosa (BN), and 28 HC. To capture the rapid dynamics of emotions, we prompted participants every 15 min over a 24-h period to assess their current perceived emotions. We analyzed group differences in terms of activation, persistence, switches, and down-regulation of emotion sequences.

**Results:**

By comparing patients with BPD to HC, we replicated five of the seven previously reported dysregulated emotion sequences, as well as 111 out of 113 unaltered sequences. However, none of the previously reported dysregulated emotion sequences exhibited specificity, i.e., none revealed higher frequencies compared to the PTSD group or the BN group. Beyond these findings, we revealed a specific finding for patients with BN, as they most frequently switched from anger to disgust.

**Conclusions:**

Replicating previously found dysregulated and unaltered emotional sequences strengthens the significance of emotion sequences. However, the lack of specificity points to emotion sequences as transdiagnostic features.

## Background

Affective dysregulation is of central importance in borderline personality disorder (BPD) as it is assumed to drive other BPD symptoms [1–3]. Much progress has been made in recent years regarding the understanding of affective dysregulation [4, 5]. Multiple studies have investigated processes such as affective instability [6–8], emotional switching [9], and emotion sequences [10] in the most important context possible, the everyday lives of patients [11]. However, there is surprisingly little evidence of specificity, namely, whether BPD patients exhibit temporal patterns of affective dysregulation distinct from other psychiatric disorders such as posttraumatic stress disorder (PTSD), bulimia nervosa (BN), major depressive disorder, and dysthymic disorder [6, 7, 9, 12]. This is especially notable given that BPD is defined as an emotionally unstable personality disorder in the ICD-10 [13].

A possible explanation is suggested by Santangelo et al. [6], who state that when examining valence, the quality of the affective states within the temporal pattern is obscured. Concretely, the emergence of anger after an affective state of shame is subsumed as a constant negative affect when considering only global valence. Empirical support for this premise is derived from the study of Trull et al. [14], who investigated the instability of certain emotions and found that patients with BPD exhibited higher instability with respect to hostility, fear, and sadness compared to patients with major depressive disorders, whereas, according to their 2008 paper, there was no significant difference regarding instability associated with negative affect. Extreme changes in hostility scores were more likely to occur in the BPD group. However, even the exploration of the course of a specific emotion lacks the information necessary to discover the quality of emotion sequences, such as the emergence of anger after an affective state of shame.

Unraveling such multi emotional patterns is only possible by investigating the activation, persistence, switch, and down-regulation of certain emotions as determined by Reisch et al. [10], who differentiated four types of emotion sequences: the activation of an emotion, the persistence of an emotion across multiple prompts, the switch from one emotion to another, and the down-regulation of an emotion. In their e-diary study, the research group identified 80 different emotion sequences resulting from eight basic emotions. The emotions of a sample of 50 patients with BPD and a sample of 50 healthy controls (HC) were assessed every 15 min over a 24-h period. Of the 80 comparisons, seven revealed significant group differences. Specifically, compared with the HC, the persistence of anxiety, the persistence of sadness, switches from sadness to anxiety, switches from anxiety to anger, and switches from anxiety to sadness were more pronounced among those in the BPD group. Conversely, the activation of joy and activation of interest occurred more frequently in the HC group.

However, as Reisch et al. [10] did not use clinical controls as comparison groups, it remains open whether these identified emotion sequences occur also with other mental disorders, i.e., whether they show specificity. For this purpose, we chose PTSD and BN as clinical control groups, because both disorders exhibited affective dysregulation in previous studies [15–18]. To our knowledge, the study of Reisch et al. [10] is the only study that investigated such emotion sequences, which is remarkable given the importance of basic emotions in Linehan’s biosocial theory [1] as well as in psychotherapy in general.

### Aims of the study

First, we aimed at replicating the findings of Reisch et al. [10], who identified seven dysregulated emotion sequences in BPD compared to HC. Accordingly, we hypothesized that patients with BPD experience the activation of joy and interest less often than HC, exhibit an increased persistence of anxiety and sadness, and have more frequent emotional switches from sadness to anxiety, from anxiety to anger, and from anxiety to sadness than HC (hypothesis 1). Second, we hypothesized that these emotion sequences are specific for BPD (hypothesis 2). For this purpose, we compared these emotion sequences in patients with BPD to patients with PTSD and BN. In a final, purely explorative step, we screened all possible variants of emotion sequences for disorder-specific differences.

## Methods

### Subjects

All patients met the DSM-IV criteria for their specific disorder. Trained postgraduate psychologists diagnosed the disorders using the German versions of the Structured Clinical Interview for DSM-IV Axis I Disorders (SCID-I) [19] and for DSM-IV Axis II Disorders (SCID-II) [20]. The inter-rater reliability of these interviews was found to be very good (Κ = 0.71 for SCID-I; Κ = 0.84 for SCID-II) [21]. Additionally, we used the BPD section of the German version of the International Personality Disorder Examination (IPDE) [22]. With respect to the patient groups, a history of schizophrenia, bipolar disorder or current substance abuse constituted exclusion criteria. Furthermore, we excluded patients from the clinical control groups who met the criteria for BPD. All other comorbidities were allowed in the clinical control groups. Lifetime or current psychiatric disorder diagnoses, psychotherapeutic treatments, and use of psychotropic medications were exclusion criteria for participation in the HC group.

Data collection of the all-female sample occurred at the Central Institute of Mental Health Mannheim and at the Psychosomatic Clinic St. Franziska Stift Bad Kreuznach in Germany. We recruited outpatients and inpatients from their outpatient clinics or wards or via advertisements in local newspapers and on the Internet. HC were selected randomly from the national resident register of the City of Mannheim or recruited via advertisement. All participants provided written informed consent prior to participation in the study, which had received prior approval from the local ethics committee.

### Assessment and data acquisition

To enable the replication of the findings of Reisch et al. [10], we used the same set of items and a similar time-based design. In previously published studies, this set of items and the chosen time-based design resulted in satisfactory methodological quality, i.e., low reactivity, high compliance, minimal patient burden, etc. (for details, see [23–25]). Participants obtained palmtop computers (Tungsten E, Palm Inc., U.S.A.) that we programmed with the DialogPad e-diary-software (Gerhard Mutz, Cologne University, Germany). After being carefully instructed in its use, participants carried the e-diary with them for a 24-h period. Every 15 min (±1 min) during their waking time, the e-diary prompted the participants, via a beep, to report their current perceived emotions. The question, “Do you feel any of the following emotions right now?” could be answered on a list composed of the following: happy, anxious, angry, shame, disgust, sad, guilt, interest, envy/jealousy, emotion but cannot name it, and no emotion. In contrast to Reisch et al. [10], we added two further emotions, guilt and jealousy, to broaden the range of emotions. If the participants selected the option “emotion but cannot name it”, they were then asked whether the current emotion was pleasant or unpleasant. In addition, participants responded to three further questions that are not reported in this manuscript. After completing the assessment period, participants handed back the devices, and the e-diary data were downloaded.

### Emotion sequences

The classification of emotion sequences is based on the procedure established by Reisch et al. [10] and was realized as follows. One emotion sequence is composed of the perceived basic emotions of two successive prompts: an emotion *E*1 at assessment point t followed by emotion *E*2 at assessment point t + 1 add up to one emotion sequence (*E*1 → *E*2). All possible variants of two consecutive emotions amount to 120 different emotion sequences. We categorized these emotion sequences into four types:

*Activation (of an emotion)*: the perception of no emotion at prompt t (*E*1) is followed by the perception of any emotion at prompt t + 1 (*E*2).

*Persistence (of an emotion)*: the perception of the same emotion in two consecutive prompts.

*Switch (a change from one emotion to another)*: the perception of any emotion is followed by the perception of a different emotion at the subsequent prompt.

*Down-regulation (of an emotion)*: the perception of any emotion is followed by the perception of no emotion at the subsequent prompt.

### Adjusted relative frequency

We followed the logic of Reisch et al. [10] to calculate the adjusted relative frequencies. However, Reisch et al. [10] used a shorter calculation method and adjusted the frequencies in relation to the group level, which was possible given that their sample sizes were identical between groups. With respect to the current data set, the sample sizes differ between groups. Therefore, we extended the adjustment to an individualized adjustment to improve accuracy.

In detail, we initially counted the frequencies of all emotion sequences (*E*1 → *E*2) for each subject. As each absolute frequency depends on the frequencies of the two contributing single emotions *E*1 and *E*2, we used the following formula to calculate an adjusted measure called the adjusted relative frequency (of the individual subject):

$$ ARF\left(E1\to E2\right)=\frac{f_S\left(E1\to E2\right)}{\ {f}_S(E1)\times {f}_S(E2)+1} $$

In the numerator, *f*_*S*_(*E*1 → *E*2) denotes the counted absolute frequency of a specific emotion sequence of the individual subject. We adjusted this absolute frequency by dividing it by the product of the individual’s frequencies of the contributing emotions *E*1 and *E*2, as represented in the denominator [*f*_*S*_(*E*1) × *f*_*S*_(*E*2)]. As an example, the number of counted emotional switches from sadness to anxiety of a single patient with BPD was divided by the product of the number of this patient’s reported feelings of sadness and anxiety. We added 1 to the product in the denominator to avoid divisions by zero in the case of non-reported emotions. We calculated the adjusted relative frequency (ARF) for each subject *S* and each sequence (*E*1 → *E*2).

Further data analysis comprised three steps: First, to replicate Reisch et al. [10], we compared the ARFs of the seven hypothesized emotion sequences between the BPD group and the HC group using t-tests for independent samples. Since the ARFs were not normally distributed but were positively skewed, we conducted nonparametric Wilcoxon rank-sum tests. To compensate for multiple testing, we reduced the alpha level from α = .05 to α = .014 via the Bonferroni correction. Second, to investigate specificity, we used Kruskal-Wallis nonparametric analysis of variance for the seven hypothesized sequences. In the case of a significant omnibus test, we used Dunn-Bonferroni post hoc tests - again setting the alpha level to .014 - to analyze group contrasts. Third, to explore any further specificity of emotion sequences, we calculated Kruskal-Wallis tests for all possible variants of emotion sequences. To limit alpha inflation, we divided the alpha level by the number of prompted emotions, thus restricting the level to .005. We contend that this ad hoc solution provides a good balance between test power and the problem of multiple comparisons. The data analysis was conducted using the software R [26] and the additional R package PMCMR [27].

## Results

### Subjects

The sample of 119 female participants was composed of 43 patients with BPD, 28 patients with PTSD, 20 patients with BN and 28 HC. Detailed sample characteristics are provided in Table 1. The mean age of the total sample was 28.6 years (range: 18 to 48). There were no significant age differences between the BPD group, the clinical controls, and the HC (Kruskal-Wallis-H = 4.15, *p* = .16). Among the three clinical groups, 42% of the patients, on average, were on psychotropic medication, on average. The most frequent comorbid current Axis I diagnoses were anxiety disorders (62%), particularly social phobia (40%), followed by major depression (37%). Comorbidity of personality disorders was highest for avoidant personality disorder (36%).

**Table 1:** Sample characteristics

Variable	BPD (*n* = 43)	PTSD (*n* = 28)	BN (*n* = 20)	HC (*n* = 28)
Age in years				
M (SD)	26.72 (7.07)	35.25 (7.53)	23.70 (5.97)	28.82 (7.47)
Variable	BPD (*n* = 43)	PTSD (*n* = 28)	BN (*n* = 20)	Χ^2^ test
Psychotropic medication				
n (%)	16 (37%)	17 (60%)	5 (25%)	PTSD > BN
Hospitalization n (%)				
Outpatients	26 (60%)	8 (29%)	9 (45%)	BPD > PTSD
Inpatients	17 (40%)	20 (71%)	11 (55%)	PTSD > BPD
Current Axis I diagnoses n (%)				
Major depression	9 (21%)	15 (54%)	10 (50%)	PTSD, BN > BPD
Anxiety disorders	27 (63%)	19 (68%)	10 (50%)	n.s.
PTSD	22 (51%)	all	3 (15%)	BPD > BN
Bulimia nervosa	9 (21%)	2 (7%)	all	n.s.
Current Axis II disorders n (%)				
Borderline	all	exclusion criterion		not applicable
Avoidant	24 (25%)	6 (21%)	3 (15%)	BPD > PTSD, BN
Obsessive-compulsive	7 (16%)	3 (11%)	2 (10%)	n.s.
Dependent	7 (16%)	0 (0%)	1 (5%)	n.s.
Paranoid	7 (16%)	3 (11%)	1 (5%)	n.s.

*BPD* borderline personality disorder, *PTSD* posttraumatic stress disorder, *BN* bulimia nervosa, *HC* healthy controls, > signals significant group differences, *n.s*, no significant group differences

### Adjusted relative frequencies of emotion sequences

**Fig. 1:**
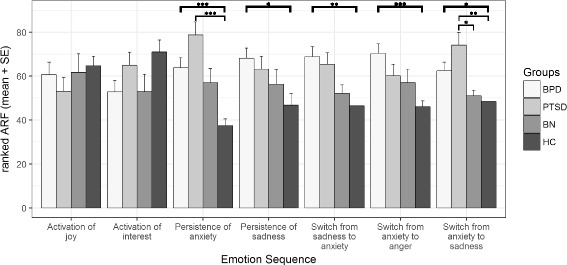
Ranks of adjusted relative frequencies of the seven hypothesized emotion sequences: means and standard errors. BPD, borderline personality disorder; PTSD, posttraumatic stress disorder; BN, bulimia nervosa; HC, healthy controls, (*) Significant group differences on the Wilcoxon rank-sum tests for hypothesis 1 regarding replication (bold print) and the Dunn-Bonferroni tests of the Kruskal-Wallis tests for hypothesis 2 regarding specificity; alpha level Bonferroni corrected (see details in the methods section)

Findings regarding the seven hypothesized emotion sequences and their specificity are presented in Fig. 1. The bars illustrate the means of the ranked ARFs, which serve as the independent variables in the nonparametric testing. Significant group differences are marked via brackets. As indicated by the brackets highlighted in bold print, we could replicate five of the seven hypothesized emotion sequences (hypothesis 1). In detail, comparing the BPD group to the HC group revealed a significantly higher frequency of persistence in anxiety (Wilcoxon rank-sum test W = 877.5, *p* < .001) and sadness (W = 808, *p* = .006) in the BPD group. Compared to the HC, patients with BPD switched more often from anxiety to sadness (W = 742, *p* = .007) and vice versa (W = 826, *p* < .001), as well as from anxiety to anger (W = 851.5, *p* < .001). No group differences could be found regarding activation of joy (W = 555, *p* = .58) and interest (W = 419, *p* = .03) after the Bonferroni correction.

However, in view of the specificity of emotion sequences in patients with BPD (hypothesis 2), none of the hypothesized differences occurred between the patients with BPD and those with PTSD or those with BN (all Dunn-Test-H-values < 2.46, all *p*-values > .08). Simply said, at the first glance, we did not find any evidence of specificity in the BPD sample. In a second step, we compared our clinical control groups to the HC. With respect to the PTSD group, we found significant group differences regarding two emotion sequences. Similar to the BPD group, the PTSD group exhibited a higher frequency of persistence in anxiety (H = 4.97, *p* < .001) compared to the HC group. In addition, the PTSD group switched more often from anxiety to sadness than did the HC group (H = 4.04, p < .001). There were no differences between the BN group and the HC group. As a third step, we compared the two clinical control groups. Data analyses revealed only one significant finding, namely, the PTSD group switched more often from anxiety to sadness in relation to the BN group (H = 3.32, *p* = .005).

**Fig. 2:**
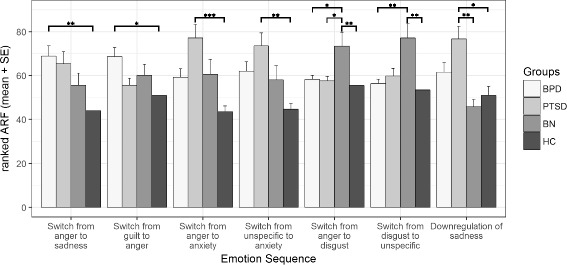
Ranks of adjusted relative frequencies: means and standard errors. BPD, borderline personality disorder; PTSD, posttraumatic stress disorder; BN, bulimia nervosa; HC, healthy controls, (*) Significant group differences on the Dunn-Bonferroni tests of the Kruskal-Wallis tests in hypothesis-free analysis; alpha level restricted to .005

In the last step, searching for disorder specific emotion sequences, we ran certain explorative, hypothesis-free analyses. As presented in Fig. 2, seven out of the remaining 113 emotional sequences showed significant group differences. Four of the sequences revealed significant differences between the HC group and one clinical disorder. That is, patients with BPD switched more often from anger to sadness (H = 3.82, *p* < .001) and from guilt to anger (H = 3.38, *p* = .004) than did the HC. Once again, no significant results between the BPD group and the clinical control groups could be found. Patients with PTSD exhibited a higher frequency of switches from anger to anxiety (H = 4.41, *p* < .001) as well as from an unspecific emotion to anxiety than did the HC (H = 3.87, *p* < .001).

In three of the emotion sequences, we found at least some evidence of specificity. Switching from anger to disgust occurred significantly more often in the BN group compared to the BPD group (H = 3.57, *p* = .002), the PTSD group (H = 3.44, *p* = .004), and the HC group (H = 3.89, *p* < .001). Furthermore, patients with BN reported more switches from disgust to an unspecific emotion compared to patients with BPD (H = 4.11, p < .001) and HC (H = 4.32, *p* < .001), but not in comparison to patients with PTSD (H = 3.17, *p* = .009). The sequence of down-regulating sadness was more common in the PTSD group compared to both the BN group (H = 3.80, p < .001) and the HC group (H = 3.46, *p* = .003).

## Discussion

This is the first study to investigate the specificity of emotion sequences in patients with BPD. As hypothesized in hypothesis 1, we replicated five of the seven results of Reisch et al. [10]. The emotion sequences classified as persistence and switch indicated significant differences between the BPD group and the HC, whereas we could not find the hypothesized differences for activation. Viewed from another perspective, the hypothesized differences occurred with reference to the emotion sequences including negative emotions, but not including positive emotions. Interestingly, the negative sequences covered the three basic emotions, i.e., anxiety, anger, and sadness. These are the same basic emotions, for which Trull et al. [14] found significant instability in his BPD e-diary study and the same negative basic emotions that are specifically listed in the BPD section of the DSM-5 [28]. Regarding the two sequences of activated positive emotions, i.e., joy and interest, revealing significant differences in the study of Reisch et al. [10], HC showed higher descriptive values than the BPD group in both cases. Furthermore, without the correction of the alpha level, the difference in activation of interest would reach significance (Cohen’s d = .53) [29], which might indicate a problem with the test power. Hence, considering that we corrected the alpha level to avoid alpha inflation, a rate of 71% of replicated results is clearly above chance and is suggestive of solid differences between patients with BPD and the HC. Further evidence for this is provided by the explorative analysis. Out of the remaining 113 comparisons of emotion sequences, only two revealed additional significant group differences between patients with BPD and the HC, which nicely maps the findings of Reisch et al. [10].

With respect to our second hypothesis, the findings were sobering. None of the seven emotion sequences of Reisch et al. [10] exhibited specificity. In two cases, the PTSD group exhibited even higher values compared to the BPD group (persistence of anxiety, switch from anxiety to sadness). In three emotion sequences, namely, the persistence of sadness, switch from sadness to anxiety, and switch from anxiety to anger, the BPD group revealed at least the highest descriptive values, and it is the only clinical group, which showed significant differences in comparison to the HC group. Nonetheless, because the effect sizes are small when comparing the BPD group to the clinical groups, we cannot assume test power to be the problem at this point. An alternative explanation could be that while the frequency of sequences does not distinguish BPD from other clinical groups, a larger magnitude of emotional intensity within the sequences will do so. Accounting for the intensities by comparing their mean changes within each of the hypothesized sequences does not, however, result in any group differences. The finding that the seven dysregulated emotion sequences cannot be attributed to a specific diagnosis implies that the emotion sequences could be transdiagnostic mechanisms, which are a topic of lively discussion in current research (e.g., [30]). In earlier daily life studies, other disorders also exhibited disturbed affective processing, such as bulimia nervosa [16–18] and posttraumatic stress disorder [15]. Similarly, concepts in psychotherapy aiming to improve emotion regulation in BPD have been adapted to the treatment of several other disorders (e.g., [31, 32]).

Regarding our purely explorative approach, we found three emotion sequences that potentially display specificity. Two of them apply to the BN group and both include disgust as a contributing emotion, namely, the switch from anger to disgust and the switch from disgust to an unspecific emotion. This is not entirely surprising given that disgust sensitivity is believed to play a role in eating disorders [33]. The finding that switches from anger to disgust are specific for BN in comparison to all other groups is excellently consistent with the study of Fox and Harrison [34], in which it was found that anger and disgust may be coupled in persons with eating pathology inasmuch as disgust may be used to manage the so called toxic emotion of anger in people with eating pathology. One might also suggest that this emotion sequence could be directly linked to the occurrence of dysfunctional eating behaviors in patients with BN. Anger-induced eating [35] could, according to the DSM-5 criteria of binge eating episodes, result in feelings of disgust [28]. To explain the second emotion sequence that showed some specificity in the BN group, i.e., the switch from disgust to an unspecific emotion, it is conceivable that after finishing a binge episode with its associated cascade of specific negative emotions, disgust may fade and leave behind unspecific negative emotions. This could be consistent with the emotion regulation model of Leehr et al. [36], which supposes that unspecific emotions play a role in the understanding of binge eating.

While the increased frequency of down-regulation of sadness in PTSD was slightly surprising, it was only partially specific. However, several studies discuss sadness as another dominant emotion in addition to anxiety in PTSD (e.g., [37, 38]). Although Power and Fyvie [37] describe a sadness-based PTSD, this ambiguous result raises open questions and warrants replication.

Summing up the findings of the explorative approach and hypothesis 2, we conclude that specific emotion sequences are an exception rather than a standard. Compared to our studies using more global measures, such as affective instability [6, 7, 9], we find some specific features, a finding that suggests a need for additional studies and replications.

The results are subject to the following methodological limitations. The sample comprises female patients only, which restricts the representativeness of the results. However, given the literature regarding sex differences and emotion [39], a pure female sample reduces heterogeneity, which may be useful. Whereas the total sample was large, subdividing it into several clinical groups limited the sample size of the subgroups. Nonetheless, having clinical control groups is a major advantage of this study. The non-significant finding for activation of interest in hypothesis 1 may be a consequence of low test power since it would have reached significance without the alpha adjustment. Nevertheless, we could replicate five of the seven sequences of Reisch et al. [10] with our given sample and with the used alpha adjustment. With respect to comorbidity, patients with BPD as well as an additional PTSD or BN diagnosis were included in the sample, whereas clinical controls were not allowed to have a comorbid BPD diagnosis. However, even after the exclusion of all patients with comorbid PTSD or BN from the BPD group in additional statistical analyses, our findings remained the same (data available upon request). Another common point against e-diary studies is the high variability in daily life. Future studies investigating emotion sequences should capture emotionally relevant events occurring during the assessment period. This would enable researchers to find connections between emotion sequences and potential trigger events. Moreover, it remains unclear whether all patient groups have the same ability to identify and specify emotions. Therefore, future research on emotion sequences could benefit from simultaneously investigating constructs such as emotional clarity [40, 41] or emotional differentiation [5]. For clinical practice, it would be of major interest whether the found emotion sequences change as a result of treatment. More specifically, future studies should investigate treatment effects of patterns of emotion sequences, i.e., whether successfully completed psychotherapy leads to a lower relative frequency of dysregulated emotion sequences in individuals with BPD. Moreover, it could be useful to directly focus certain emotion regulation strategies, e.g., from the DBT skills training [42], on emotion sequences dysregulated in BPD.

## Conclusions

In summary, patients with BPD were more often trapped between feelings of anxiety and sadness, more often oscillated between anxiety and sadness, and more often experienced anxiety prior to experiencing anger in comparison to HC. By confirming, in large part, the findings of Reisch et al. [10], we conducted a successful replication study. Our findings indicate robust differences between patients with BPD and HC and strengthen the significance of emotion sequences. However, we did not find distinct specificity of emotion sequences in patients with BPD compared to other patient groups, namely, patients with PTSD and patients with BN. The lack of specificity suggests that these emotion sequences could be transdiagnostic features. Nonetheless, finding the first evidence of disorder specific emotion sequences in the BN group, we deem emotion sequences a promising approach to investigate affective dysregulation. Future studies should address whether emotion sequences change as a result of treatment in the patient groups.

## Abbreviations

ARF: Adjusted relative frequency
BN: Bulimia nervosa
BPD: Borderline personality disorder
HC: Healthy controls
IPDE: International personality disorder examination
PTSD: Posttraumatic stress disorder
SCID-I: Structured Clinical Interview for DSM-IV Axis I Disorders
SCID-II: Structured Clinical Interview for DSM-IV Axis II Disorders

## References

[1] Linehan M (1993). Cognitive-behavioral treatment of borderline personality disorder.

[2] Siever LJ, Torgersen S, Gunderson JG, Livesley W, Kendler KS (2002). The borderline diagnosis III: identifying endophenotypes for genetic studies. Biol Psychiatry.

[3] Tragesser SL, Solhan M, Schwartz-Mette R, Trull TJ (2007). The role of affective instability and impulsivity in predicting future BPD features. J Personal Disord.

[4] Santangelo P, Bohus M, Ebner-Priemer UW (2014). Ecological momentary assessment in borderline personality disorder: a review of recent findings and methodological challenges. J Personal Disord.

[5] Trull TJ, Lane SP, Koval P, Ebner-Priemer UW (2015). Affective dynamics in psychopathology. Emot Rev.

[6] Santangelo P, Reinhard I, Mussgay L, Steil R, Sawitzki G, Klein C (2014). Specificity of affective instability in patients with borderline personality disorder compared to posttraumatic stress disorder, bulimia nervosa, and healthy controls. J Abnorm Psychol.

[7] Santangelo PS, Limberger MF, Stiglmayr C, Houben M, Coosemans J, Verleysen G (2016). Analyzing subcomponents of affective dysregulation in borderline personality disorder in comparison to other clinical groups using multiple e-diary datasets. Borderline Personal Disord Emot Dysregul.

[8] Ebner-Priemer UW, Houben M, Santangelo P, Kleindienst N, Tuerlinckx F, Oravecz Z (2015). Unraveling affective dysregulation in borderline personality disorder: a theoretical model and empirical evidence. J Abnorm Psychol.

[9] Houben M, Bohus M, Santangelo PS, Ebner-Priemer U, Trull TJ, Kuppens P (2016). The specificity of emotional switching in borderline personality disorder in comparison to other clinical groups. Pers Disord.

[10] Reisch T, Ebner-Priemer UW, Tschacher W, Bohus M, Linehan MM (2008). Sequences of emotions in patients with borderline personality disorder. Acta Psychiatr Scand.

[11] Trull TJ, Ebner-Priemer U (2014). The role of ambulatory assessment in psychological science. Curr Dir Psychol Sci.

[12] Kohling J, Moessner M, Ehrenthal JC, Bauer S, Cierpka M, Kammerer A (2016). Affective instability and reactivity in depressed patients with and without borderline pathology. J Personal Disord.

[13] World Health Organisation (1992). The ICD-10 classification of mental and behavioural disorders: clinical descriptions and diagnostic guidelines.

[14] Trull TJ, Solhan MB, Tragesser SL, Jahng S, Wood PK, Piasecki TM, Watson D (2008). Affective instability: measuring a core feature of borderline personality disorder with ecological momentary assessment. J Abnorm Psychol.

[15] Kashdan TB, Uswatte G, Steger MF, Julian T (2006). Fragile self-esteem and affective instability in posttraumatic stress disorder. Behav Res Ther.

[16] Anestis MD, Selby EA, Crosby RD, Wonderlich SA, Engel SG, Joiner TE (2010). A comparison of retrospective self-report versus ecological momentary assessment measures of affective lability in the examination of its relationship with bulimic symptomatology. Behav Res Ther.

[17] Selby EA, Doyle P, Crosby RD, Wonderlich SA, Engel SG, Mitchell JD, Le Grange D (2012). Momentary emotion surrounding bulimic behaviors in women with bulimia nervosa and borderline personality disorder. J Psychiatr Res.

[18] Vansteelandt K, Probst M, Pieters G (2013). Assessing affective variability in eating disorders: affect spins less in anorexia nervosa of the restrictive type. Eat Behav.

[19] Wittchen HU, Wunderlich U, Gruschwitz S, Zaudig M (1997). SKID I. Strukturiertes Klinisches Interview für DSM-IV. Achse I: Psychische Störungen. Interviewheft und Beurteilungsheft. Eine deutschsprachige, erweiterte Bearbeitung der amerikanischen Originalversion des SCID-I.

[20] Fydrich T, Renneberg B, Schmitz B, Wittchen HU (1997). SKID II. Strukturiertes Klinisches Interview für DSM-IV, Achse II: Persönlichkeitsstörungen. Interviewheft. Eine deutschsprachige, erweiterte Bearbeitung der amerikanischen Originalversion des SCID-I.

[21] Lobbestael J, Leurgans M, Arntz A (2011). Inter-rater reliability of the structured clinical interview for DSM-IV Axis I disorders (SCID I) and Axis II disorders (SCID II). Clin Psychol Psychother..

[22] Mombour W, Zaudig M, Berger P, Gutierrez K, Berner W, Berger K (1996). International personality disorder examination (IPDE).

[23] Ebner-Priemer UW, Kuo J, Kleindienst N, Welch SS, Reisch T, Reinhard I (2007). State affective instability in borderline personality disorder assessed by ambulatory monitoring. Psychol Med.

[24] Ebner-Priemer UW, Sawitzki G (2007). Ambulatory assessment of affective instability in borderline personality disorder. Eur J Psychol Assess.

[25] Ebner-Priemer UW, Welch SS, Grossman P, Reisch T, Linehan MM, Bohus M (2007). Psychophysiological ambulatory assessment of affective dysregulation in borderline personality disorder. Psychiatry Res.

[26] R Core Team. R: A language and environment for statistical computing. R foundation for statistical computing. Vienna: 2017. https://www.R-project.org. Assessed 1 Sept 2017.

[27] Pohlert T. The Pairwise multiple comparison of mean ranks package (PMCMR). R package; 2014. http://CRAN.R-project.org/package=PMCMR. Assessed 1 Sept 2017.

[28] American Psychiatric Association (2013). Diagnostic and statistical manual of mental disorders.

[29] Cohen J (1988). Statistical power analysis for the behavioral sciences.

[30] Aldao A (2016). Introduction to the special issue: emotion regulation as a Transdiagnostic process. Cognit Ther Res.

[31] Roosen MA, Safer D, Adler S, Cebolla A, van Strien T (2012). Group dialectical behavior therapy adapted for obese emotional eaters; a pilot study. Nutr Hosp.

[32] Steil R, Dyer A, Priebe K, Kleindienst N, Bohus M (2011). Dialectical behavior therapy for posttraumatic stress disorder related to childhood sexual abuse: a pilot study of an intensive residential treatment program. J Trauma Stress.

[33] Troop NA, Murphy F, Bramon E, Treasure JL (2000). Disgust sensitivity in eating disorders: a preliminary investigation. Int J Eat Disord.

[34] Fox JRE, Harrison A (2008). The relation of anger to disgust: the potential role of coupled emotions within eating pathology. Clin Psychol Psychother.

[35] Appelhans BM, Whited MC, Schneider KL, Oleski J, Pagoto SL (2011). Response style and vulnerability to anger-induced eating in obese adults. Eat Behav.

[36] Leehr EJ, Krohmer K, Schag K, Dresler T, Zipfel S, Giel KE (2015). Emotion regulation model in binge eating disorder and obesity -- a systematic review. Neurosci Behav Rev.

[37] Power MJ, Fyvie C (2013). The role of emotion in PTSD: two preliminary studies. Behav Cogn Psychother.

[38] Hathaway LM, Boals A, Banks JB (2010). PTSD symptoms and dominant emotional response to a traumatic event: an examination of DSM-IV criterion A2. Anxiety Stress Coping.

[39] Fischer A (2000). Gender and emotion: social psychological perspectives.

[40] Lischetzke T, Eid M. The functionality of emotional clarity: a process-oriented approach to understanding the relation between emotional clarity and well-being. In: Robinson MD, Eid M, editors. The happy mind: cognitive contributions to well-being. New York: Springer. in press

[41] Lischetzke T, Angelova R, Eid M (2011). Validating an indirect measure of clarity of feelings: evidence from laboratory and naturalistic settings. Psychol Assess.

[42] Linehan M (2014). DBT skills training manual.

